# Mid-Regional Pro-Adrenomedullin in Combination With Pediatric Early Warning Scores for Risk Stratification of Febrile Children Presenting to the Emergency Department: Secondary Analysis of a Nonprespecified United Kingdom Cohort Study*

**DOI:** 10.1097/PCC.0000000000003075

**Published:** 2022-10-14

**Authors:** Rebecca A. F. Lenihan, Juliana Ang, Philip Pallmann, Sam T. Romaine, Cherry-Ann Waldron, Emma Thomas-Jones, Nahida Miah, Enitan D. Carrol

**Affiliations:** 1 Department of Clinical Infection, Microbiology and Immunology, University of Liverpool, Institute of Infection, Veterinary and Ecological Sciences, Liverpool, United Kingdom.; 2 Centre for Trials Research, College of Biomedical & Life Sciences, Cardiff University, Cardiff, United Kingdom.

**Keywords:** adrenomedullin, Early Warning Score, emergency department, pediatrics, procalcitonin, risk stratification

## Abstract

Current sepsis guidelines do not provide good risk stratification of subgroups in whom prompt IV antibiotics and fluid resuscitation might of benefit. We evaluated the utility of mid-regional pro-adrenomedullin (MR-proADM) in identification of patient subgroups at risk of requiring PICU or high-dependency unit (HDU) admission or fluid resuscitation.

**DESIGN:**

Secondary, nonprespecified analysis of prospectively collected dataset.

**SETTING:**

Pediatric Emergency Department in a United Kingdom tertiary center.

**PATIENTS:**

Children less than 16 years old presenting with fever and clinical indication for venous blood sampling (*n* = 1,183).

**INTERVENTIONS:**

None.

**MEASUREMENTS AND MAIN RESULTS:**

Primary outcome measures were PICU/HDU admission or administration of fluid resuscitation, with a secondary outcome of definite or probable bacterial infection. Biomarkers were measured on stored plasma samples and children phenotyped into bacterial and viral groups using a previously published algorithm. Of the 1,183 cases, 146 children (12.3%) required fluids, 48 (4.1%) were admitted to the PICU/HDU, and 244 (20.6%) had definite or probable bacterial infection. Area under the receiver operating characteristic (AUC) was used to assess performance. MR-proADM better predicted fluid resuscitation (AUC, 0.73; 95% CI, 0.67–0.78), than both procalcitonin (AUC, 0.65; 95% CI, 0.59–0.71) and Pediatric Early Warning Score (PEWS: AUC, 0.62; 95% CI, 0.56–0.67). PEWS alone showed good accuracy for PICU/HDU admission 0.83 (0.78–0.89). Patient subgroups with high MR-proADM (≥ 0.7 nmol/L) and high procalcitonin (≥ 0.5 ng/mL) had increased association with PICU/HDU admission, fluid resuscitation, and bacterial infection compared with subgroups with low MR-proADM (< 0.7 nmol/L). For children with procalcitonin less than 0.5 ng/mL, high MR-proADM improved stratification for fluid resuscitation only.

**CONCLUSIONS:**

High MR-proADM and high procalcitonin were associated with increased likelihood of subsequent disease progression. Incorporating MR-proADM into clinical risk stratification may be useful in clinician decision-making regarding initiation of IV antibiotics, fluid resuscitation, and escalation to PICU/HDU admission.

**RESEARCH IN CONTEXT**

- Although 41% of febrile children present to the pediatric ED with warning signs of sepsis, few end up undergoing investigation or treatment for true sepsis (9).
- There is growing evidence that MR-proADM and procalcitonin are useful predictors of sepsis and septic shock in pediatric populations.
- Use of biomarkers, alongside clinical severity scores such as PEWS, could improve risk stratification and support clinical decision-making, leading to more judicious use of antibiotics, fluid resuscitation, and PICU/HDU escalation, without unnecessarily treating large numbers of children with self-limiting infection.

Febrile illness is the second most common medical presentation to the pediatric emergency department (ED) (1). Most of these children will not have life-threatening bacterial infection but those who do are difficult to differentiate clinically from those with a self-limiting viral illness. U.K. National Institute for Health and Care Excellence (NICE) guidelines recommend senior review within 1 hour for children meeting NICE high-risk sepsis criteria; in a recent study of febrile children presenting to ED, 55% of children met these criteria (2). The intervention cost of broad screening algorithms are therefore significant, especially given that invasive infections are infrequent, and the majority of febrile children presenting to ED can be appropriately managed conservatively without further escalation (3). Additionally, 32% of febrile children presenting to ED receive antibiotics, despite only 7% of these later being confirmed to have a bacterial cause of their symptoms (4). This problem fuels rising rates of antimicrobial resistance, as well as increased risk of adverse effects from antibiotics and fluid overload. To standardize the approach to the unwell child and improve patient safety, a U.K. National Pediatric Early Warning Score (N-PEWS) is being developed and evaluated in United Kingdom (5, 6) and has been shown to be a good predictor for PICU or high-dependency unit (HDU) admission (7) in children presenting to the ED.

Adrenomedullin is a vasodilator peptide involved in regulation of endothelial function (8, 9) in sepsis and septic shock (10, 11). Adrenomedullin has a short half-life (12), therefore, its derivative, mid-regional pro-adrenomedullin (MR-proADM) is measured, as it directly correlates with levels of adrenomedullin in the circulation (13). MR-proADM has demonstrated significant clinical utility in risk stratification in adults (14) with suspected infection in the ED (15, 16) and for prediction of mortality in intensive care (17). MR-proADM was useful in evaluating severity and prognosis of sepsis (18) and community acquired pneumonia (19) in children. Procalcitonin, a precursor to the hormone calcitonin, is typically undetectable in the serum of healthy patients, but it is expressed in significantly higher concentrations with bacterial infection (18, 20). Levels of procalcitonin are proportional to both frequency of complications and to treatment response (11, 18, 20).

The aim of this study was to determine whether MR-proADM level at presentation, in combination with clinical severity scores (PEWS) and biomarkers of bacterial infection (procalcitonin), could stratify for disease severity, allowing identification of children likely to require IV antibiotics, fluid resuscitation or PICU/HDU admission, enabling initiation of appropriate treatment at the earliest opportunity.

## MATERIALS AND METHODS

This study was a secondary, nonprespecified analysis of a previously reported observational study of febrile children presenting to ED (21). Data were originally collected from febrile children with a clinical indication for blood sampling presenting to the Alder Hey (AH) Children’s Hospital ED in Liverpool, United Kingdom, between November 1, 2010, and April 3, 2012 (21). There were no prespecified criteria for blood sampling; decisions regarding blood sampling, laboratory investigations, and bacterial and viral testing were made by the treating clinical team at time of triage. Approval for the study was granted by the Greater Manchester West Research Ethics Committee (10/H1014/53) and by the AH Children’s Hospital Research and Development department.

### Data Collection

The original study included children under 16 years old presenting consecutively with either a fever of greater than 38°C or history of fever in the preceding 24 hours (21). Independent secondary data analysis was performed on the previously collected database of eligible ED attendances (21). PEWS were calculated retrospectively from the first recorded observations at triage and biomarkers measured retrospectively on stored plasma samples. N-PEWS and AH-PEWS and were calculated retrospectively from the electronic database as per recently published criteria (7). Specifically, “work of breathing” was classified as mild in N-PEWS, with “stridor” and “cyanosis” classed as severe. Any missing parameters were assumed to be normal (7). Although clinicians had access to AH-PEWS at time of patient assessment, clinical decisions were made blind to MR-proADM and procalcitonin results. Conversely, the researchers performing the MR-proADM and procalcitonin analysis were blinded to PEWS and clinical details. Where data were missing, children were not excluded from analysis but were included if the overall PEWS could be calculated and there was sufficient stored blood sample for secondary analysis.

AH critical care unit (CCU) covers three areas: ICU, high-dependency unit, and the burns unit. There are 48 beds in the CCU. The 15-bed HDU supports children requiring detailed observations and interventions or single organ support. Critical care was defined as admission to PICU/HDU within 48 hours of admission, ensuring that children who were transferred from ED to another hospital ward and subsequently deteriorated were not missed. Fluid resuscitation was defined as administration of IV saline, fresh frozen plasma, or albumin within 6 hours of admission to ED. Children were phenotyped into bacterial and viral groups using a previously published algorithm (22).

### Samples

Blood samples were taken at admission to ED and handled as previously described (21). MR-proADM levels were analyzed on archived plasma samples, stored at –80°C, using the commercially available automated fluorescence sandwich immunoassay B.R.A.H.M.S. Kryptor (Thermo Fisher Scientific, Raleigh, NC) in accordance with manufacturer’s instructions.

### Data Analysis

Data analysis was performed using SPSS Version 28 (IBM SPSS Statistics, IBM Corporation) and R Version Corporation, Armonk, NY) and R Version 4.1.0 (R Foundation for Statistical Computing, Vienna, Austria; https://www.r-project.org/). Optimal cutoffs for PEWS (7), MR-proADM, and procalcitonin (15) were identified using Youden’s J statistic (23). High N-PEWS was defined as greater than or equal to 6, high AH-PEWS as greater than or equal to 3, high procalcitonin as greater than or equal to 0.5 ng/mL, and high MR-proADM as greater than or equal to 0.7 nmol/L. Primary outcome measures were need for PICU/HDU admission or fluid resuscitation, with a secondary outcome measure of definite or probable bacterial infection. For assessment of PEWS, MR-proADM and procalcitonin in differentiating bacterial and viral infection, bacterial infection was defined as children meeting criteria for definite or probable bacterial infection, and viral infection as children meeting criteria of definite or probable viral infection (**Supplementary Fig. 1**, http://links.lww.com/PCC/C221; **legend**, http://links.lww.com/PCC/C229) (22). As the phenotyping case definition included C-reactive protein (CRP), it was not appropriate to assess its performance in differentiating bacterial and viral infection. Receiver operating characteristic curves were generated individually for PEWS, MR-proADM, procalcitonin, and CRP, then for AH- and N-PEWS with procalcitonin greater than or equal to 0.5 ng/mL and MR-proADM greater than or equal to 0.7 nmol/L individually, then with both biomarker thresholds combined (**Supplementary Fig. 2**, http://links.lww.com/PCC/C222; legend, http://links.lww.com/PCC/C229). Logistic regressions were performed to assess whether combining AH-PEWS greater than or equal to 3 or N-PEWS greater than or equal to 6 with either a procalcitonin of greater than or equal to 0.5 ng/mL, an MR-proADM of greater than or equal to 0.7 nmol/L, or both, improved the area under the receiver operating characteristic (AUC) for PICU/HDU admission, fluid resuscitation, and definite or probable bacterial infection.

The original study was reported in line with Standards for Reporting of Diagnostic Accuracy and Transparent Reporting of a multivariable prediction model for Individual Prognosis Or Diagnosis guidelines (21, 24, 25). This includes the method of recruitment of patients, order of test execution, number of patients undergoing the tests under evaluation and the numbers of patients with the reference standard.

## RESULTS

There were 1,183 children meeting inclusion criteria (**Supplementary Fig. 3**, http://links.lww.com/PCC/C223; legend, http://links.lww.com/PCC/C229) (21); 55% were male and one child died. The median age was 2.5 years old, including 21 neonates and the youngest patient was 7 days old. A total of 146 children (12.3%) required fluids, and 48 (4.1%) were admitted to PICU/HDU. The mean time from admission to blood was 2 hours and 12 minutes (sd 1 hr 18 min), with a maximum time of 8 hours and 38 minutes. Measurements were available for MR-proADM in 792 children, procalcitonin in 1,106 children, and CRP in 1,151 children (**Supplementary Table 1**, http://links.lww.com/PCC/C224). Missing PEWS data are summarized in **Supplementary Table 2** (http://links.lww.com/PCC/C225). Children were phenotyped according to the algorithm (22) (Supplementary Fig. 1, http://links.lww.com/PCC/C221; legend, http://links.lww.com/PCC/C229): 77 (6.5%) had definite bacterial infection; 167 (14.1%) probable bacterial infection; 92 (7.8%) definite viral infection; and 539 (45.6%) probable viral infection. Of 1,183 children, 359 (30.3%) had at least one preexisting comorbidity and 106 (9.0%) had multiple comorbidities (**Supplementary Table 3**, http://links.lww.com/PCC/C226). There was no significant difference in mean MR-proADM between children with a preexisting cardiac, renal, or endocrine comorbidity and children who were previously fit and well (Table 1). Analysis of the neonatal subgroup (*n* = 13) demonstrated an association between age less than or equal to 28 days and a significantly higher mean MR-proADM than the remaining population (Table 1).

**TABLE 1.** Comparison of Mean Mid-Regional Pro-Adrenomedullin Values Inpatient Comorbidity Subgroups

Subgroup	No. of Patients	Mean MR-proADM in Subgroup (nmol/L)	Mean MR-proADM in Remaining Population (nmol/L)	Mean Difference (95% CI) (nmol/L)
Cardiac comorbidity	13/792	0.69	0.57	–0.12 (–0.30 to 0.07)
Renal comorbidity	11/792	0.51	0.57	0.06 (–0.14 to 0.26)
Endocrine comorbidity	11/792	0.57	0.57	0.00 (–0.20 to 0.20)
Neonate	13/792	0.78	0.57	–0.22 (–0.40 to –0.04)

MR-proADM = mid-regional pro-adrenomedullin.

### PICU/HDU Admission

**Figure 1.**
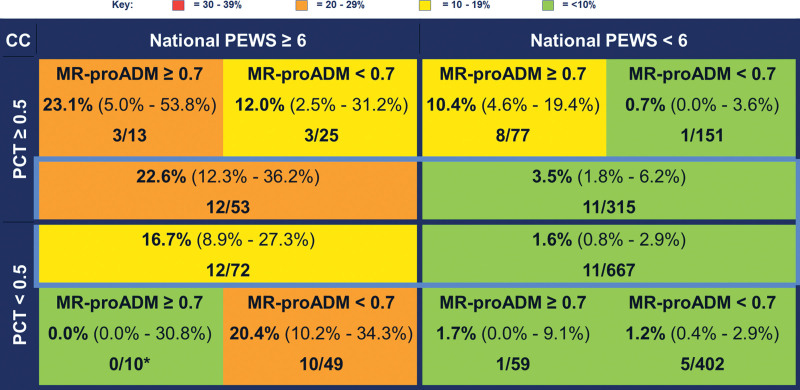
Risk stratification by mid-regional pro-adrenomedullin (MR-proADM), procalcitonin (PCT), and National Pediatric Early Warning Score (PEWS) for critical care (CC) admission. Values stated are absolute percentages (with 95% CIs) with frequencies of children with each given outcome over total number of children meeting stated MR-proADM, PCT, and National PEWS criteria. Groups for risk stratification highlighted in *light blue* include all children with a PCT and PEWS value available, with the outer substratification *boxes* only including children with an available PCT, PEWS, and MR-proADM with no missing values.

Regarding admission to PICU/HDU, AH-PEWS (AUC, 0.88; 95% CI, 0.84–0.92) and N-PEWS (AUC, 0.83; 95% CI, 0.78–0.89) both showed very good discrimination (Table 2). MR-proADM (AUC, 0.62; 95% CI, 0.51–0.73), procalcitonin (AUC, 0.57; 95% CI, 0.46–0.68), and CRP (AUC, 0.58; 95% CI, 0.50–0.664) performed less well. The AUC for procalcitonin and N-PEWS combined was 0.84 (95% CI, 0.78–0.90) and for MR-proADM and N-PEWS combined was 0.85 (95% CI, 0.78–0.92). However, the addition of procalcitonin to the combined N-PEWS and MR-proADM did not further improve discrimination (AUC, 0.85; 95% CI, 0.78–0.92). For children with PEWS less than 6 and procalcitonin of greater than or equal to 0.5 ng/mL, the pre-test probability of admission to PICU/HDU was 3.5% (95% CI, 1.8–6.2%) (Fig. 1). However, children with MR-proADM of greater than or equal to 0.7 nmol/L have a higher post-test probability of admission to HDU/PICU than those with MR-proADM less than 0.7 nmol/L (10.4% [95% CI, 4.6–19.4%] vs 0.7% [95% CI, 0.0%–3.6%], respectively) (Fig. 1). In children with both N-PEWS greater than or equal to 6 and procalcitonin greater than or equal to 0.5 ng/mL, the pre-test probability of HDU/PICU admission was 22.6% (95% CI, 12.3–36.2%) (Fig. 1). For children with MR-proADM greater than or equal to 0.7 nmol/L, the post-test probability of HDU/PICU admission is comparable (23.1%; 95% CI, 5.0–53.8%) (Fig. 1). However, for children with MR-proADM less than 0.7 nmol/L, this risk was halved, with a post-test probability of 12.0% (95% CI, 2.5–31.2%) (Fig. 1). In children with both procalcitonin less than 0.5 ng/mL and N-PEWS less than 6, there was no significant difference between the pre-test probability (1.6%; 95% CI, 0.8–2.9%), and the post-test probability for children with MR-proADM greater than or equal to 0.7 nmol/L (1.7%; 95% CI, 0.0–9.1%), or MR-proADM less than 0.7 nmol/L (1.2%; 95% CI, 0.4–2.9%) (Fig. 1).

**TABLE 2.** Area Under the Receiver Operating Curves for Fluid Resuscitation, Critical Care Admission, and Two Bacterial and Viral Infection Phenotyping Subgroups

Biomarker/Clinical Score (Threshold)	Critical Care Admission Within 48 hr (95% CI)	Fluid Resuscitation (95% CI)	Definite and Probable Bacterial vs Definite and Probable Viral Infection (95% CI)	Definite Bacterial vs Definite Viral Infection (95% CI)
Alder Hey PEWS (≥ 3)	0.88 (0.84–0.92)	0.65 (0.60–0.69)	0.54 (0.50–0.58)	0.35 (0.27–0.43)
National PEWS (≥ 6)	0.83 (0.78–0.89)	0.67 (0.62–0.71)	0.50 (0.46–0.55)	0.40 (0.32–0.49)
MR-proADM (≥ 0.70 nmol/L)	0.62 (0.51–0.73)	0.72 (0.67–0.78)	0.63 (0.58–0.67)	0.63 (0.53–0.73)
Procalcitonin (≥ 0.50 ng/mL)	0.57 (0.46–0.68)	0.67 (0.62–0.72)	0.78 (0.74–0.82)	0.77 (0.69–0.84)
C-reactive protein (≥ 20 mg/dL)	0.58 (0.50–0.66)	0.57 (0.52–0.63)	NA	NA
National PEWS (≥ 6) and procalcitonin (≥ 0.50 ng/mL)	0.84 (0.78–0.90)	0.71 (0.66–0.75)	0.77 (0.74–0.81)	0.83 (0.77–0.89)
National PEWS (≥ 6) and MR-proADM (≥ 0.70 nmol/L)	0.85 (0.78–0.92)	0.73 (0.68–0.79)	0.64 (0.59–0.68)	0.71 (0.63–0.80)
National PEWS (≥ 6) and procalcitonin (≥ 0.50 ng/mL) and MR-proADM (≥ 0.70 nmol/L)	0.85 (0.78–0.92)	0.74 (0.68–0.79)	0.73 (0.68–0.77)	0.83 (0.76–0.90)

MR-proADM = mid-regional pro-adrenomedullin, NA = not available, PEWS = Pediatric Early Warning Score.

### Fluid Resuscitation

Fluid resuscitation was documented for the first 6 hours of admission; volumes administered ranged between 30 and 1,760 mL. Of the 146 children administered fluids, 37 children received fluids prior to blood sampling, with an average time of 105 minutes from fluid administration to blood sampling. The mean MR-proADM for children receiving fluid resuscitation prior blood sampling was not significantly different from those receiving fluid after blood sampling (mean difference, –0.07; 95% CI, –0.26 to 0.11). MR-proADM was the best predictor of fluid resuscitation of the (AUC, 0.72; 95% CI, 0.67–0.78) (Table 2). Procalcitonin (AUC, 0.67; 95% CI, 0.62–0.72), N-PEWS (AUC, 0.67; 95% CI, 0.62–0.71), and CRP (AUC, 0.57; 95% CI, 0.52–0.63) showed moderate discrimination for fluid resuscitation.

**Figure 2.**
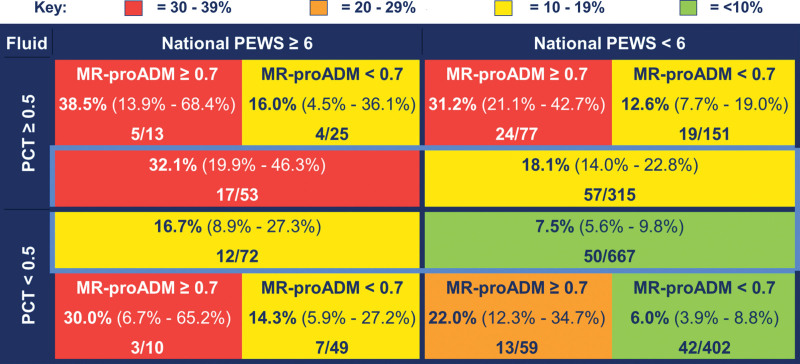
Risk stratification by mid-regional pro-adrenomedullin (MR-proADM), procalcitonin (PCT) and National Pediatric Early Warning Score (PEWS) for fluid resuscitation. Values stated are absolute percentages (with 95% CIs) with frequencies of children with each given outcome over total number of children meeting stated MR-proADM, PCT, and National PEWS criteria. Groups for risk stratification highlighted in *light blue* include all children with a PCT and PEWS value available, with the outer substratification *boxes* only including children with an available PCT, PEWS, and MR-proADM with no missing values.

Across all groups risk stratified by N-PEWS and procalcitonin (Fig. 2), the post-test probability of fluid resuscitation was higher in those with MR-proADM greater than or equal to 0.7 nmol/L than those with MR-proADM less than 0.7 nmol/L (Fig. 2). For children with N-PEWS greater than or equal to 6 and procalcitonin greater than or equal to 0.5 ng/mL, the pre-test probability of fluid resuscitation was 32.1% (95% CI, 19.9–46.3%). In this group, the post-test probability of fluid resuscitation was double in children with MR-proADM greater than or equal to 0.7 nmol/L (38.5%; 95% CI, 13.9–68.4%) compared with those with MR-proADM less than 0.7 nmol/L (16%; 95% CI, 4.5–36.1%). In children with N-PEWS less than 6 and procalcitonin less than 0.5 ng/mL, the pre-test probability of requiring fluid resuscitation was 7.5% (95% CI, 5.6–9.8%), which is comparable to the post-test probability in children with MR-proADM less than 0.7 nmol/L (6.0%; 95% CI, 3.9%–8.8%) but significantly lower than in children with MR-proADM greater than or equal to 0.7 nmol/L (22%; 95% CI, 12.3–34.7%).

### Bacterial and Viral Infection

**Figure 3.**
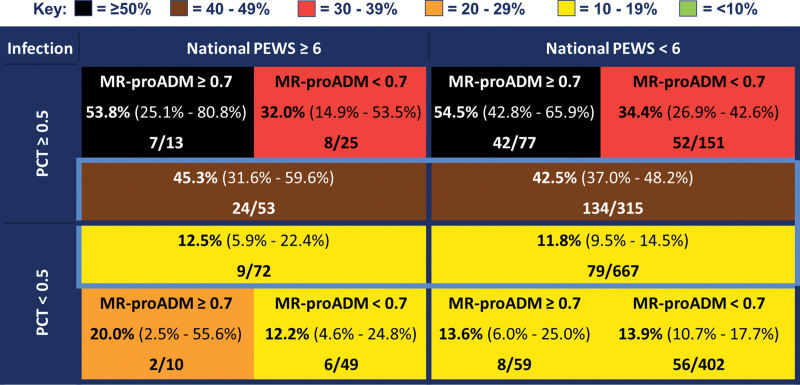
Risk stratification by mid-regional pro-adrenomedullin (MR-proADM), procalcitonin (PCT), and National Pediatric Early Warning Score (PEWS) for definite and probable bacterial versus definite and probable viral infection. Values stated are absolute percentages (with 95% CIs) with frequencies of children with each given outcome over total number of children meeting stated MR-proADM, PCT, and National PEWS criteria. Groups for risk stratification highlighted in *light blue* include all children with a PCT and PEWS value available, with the outer substratification *boxes* only including children with an available PCT, PEWS, and MR-proADM with no missing values.

Procalcitonin showed good discrimination between definite bacterial and definite viral infection (AUC, 0.77; 95% CI, 0.69–0.84), MR-proADM showed moderate discrimination (AUC, 0.63; 95% CI, 0.53–0.73), but N-PEWS (AUC, 0.40; 95% CI, 0.32–0.49) and AH-PEWS (AUC, 0.35; 95% CI, 0.27–0.43) performed poorly. The results were similar for discrimination between definite and probable bacterial infection versus definite and probable viral infection (Table 2). For children with procalcitonin greater than or equal to 0.5 ng/mL, the post-test probability of bacterial infection was 1.5 times higher in children with MR-proADM greater than or equal to 0.7 nmol/L than those with less than 0.7 nmol/L (Fig. 3). For children with both procalcitonin greater than or equal to 0.5 ng/mL and MR-proADM greater than or equal to 0.7 nmol/L, the post-test probability of bacterial infection was over 50%, irrespective of PEWS. For children with low procalcitonin (< 0.5 ng/mL), the pre- and post-test probabilities for stratification by MR-proADM were largely unchanged (Fig. 3). Risk stratification including only patients with definite bacterial or viral infection is shown in **Supplementary Figure 4** (http://links.lww.com/PCC/C227; legend, http://links.lww.com/PCC/C229).

## DISCUSSION

MR-proADM measurement is associated with improved risk stratification of febrile children attending ED and, combined with procalcitonin and N-PEWS, could guide clinical decision-making, including administration of urgent IV antibiotics, fluid resuscitation and PICU/HDU escalation.

**AT THE BEDSIDE**

- In our study, PEWS was the best predictor of PICU/HDU admission, MR-proADM was the best predictor of fluid resuscitation, and procalcitonin was the best discriminator between bacterial and viral infection.
- We demonstrated substratification by MR-proADM in febrile children at the time of presentation improves identification of children at risk of fluid resuscitation, bacterial infection, and PICU/HDU admission.
- Incorporation of MR-proADM, procalcitonin and PEWS into a risk-stratification algorithm could guide clinical decision-making in febrile children attending ED, including administration of urgent IV antibiotics, fluid resuscitation, and escalation to PICU/HDU.

MR-proADM measured in children presenting to ED with fever, when compared with CRP, procalcitonin and PEWS, is the single strongest predictor for need for fluid resuscitation within 6 hours. When substratifying by MR-proADM alongside procalcitonin and PEWS, a high MR-proADM was associated with increased post-test probability of fluid resuscitation across all groups, regardless of procalcitonin and PEWS. Our data are consistent with published studies, which demonstrated that MR-proADM enhanced mortality risk stratification in adults with suspected infection presenting to ED (15) and has prognostic value in sepsis (9, 18, 26).

Hypertension (27), heart failure (28), and renal failure (27) are associated with increased adrenomedullin levels in adults, with evidence of increased MR-proADM expression in pediatric heart failure (29). In our study, however, there was no significant difference in mean MR-proADM between patient subgroups with preexisting renal, cardiac, and endocrine comorbidities and those without. Previous evidence suggests that MR-proADM is higher at birth (7) and that higher reference ranges should be used in neonates than in adults (8). Our study demonstrates that children in the late neonatal period had a significantly higher MR-proADM than the remaining study population.

Procalcitonin was the best predictor of bacterial infection, however, the post-test probability of bacterial infection was higher for children with MR-proADM greater than or equal to 0.7 mmol/L for all groups except those with both a low PEWS and a low procalcitonin. The AUCs for National and AH-PEWS for discriminating definite bacterial and definite viral infection were notably poor. This is not unexpected; PEWS were developed for the prediction of clinical deterioration of children, rather than differentiation between bacterial and viral infection.

This study was a secondary analysis of a prospective single-center study in a high-income ED setting, with a low sepsis prevalence in the general population but a high proportion of children with complex comorbidities (~30% had a preexisting comorbidity and 9% had multiple comorbidities). Criteria for admission to PICU/HDU vary with local practice; larger, multicenter studies, including EDs with a high sepsis prevalence, would therefore be important for validation of these results.

Our study only included children in whom clinicians felt blood tests were indicated on review in the ED, so the results will apply to that selected population. There were 7 years between sample collection and MR-proADM analysis, but as there were no other freeze-thaw cycles, we do not believe that sample quality was affected.

Missing vital sign data poses a significant problem for studies evaluating PEWS, especially in an ED setting, and is a limitation of our study. However, risk stratification figures generated excluding patients with missing data demonstrate that the impact on our results was minimal (**Supplementary Fig. 5**, http://links.lww.com/PCC/C228; legend, http://links.lww.com/PCC/C229). The most common missing parameter in our study is blood pressure. NICE do not recommend routine blood pressure measurement in febrile children in the absence of abnormal capillary refill time (CRT) or heart rate (30). A study of febrile children in European EDs demonstrated that in the presence of documented abnormal CRT or heart rate, blood pressure was still missing in 77% of children (31), reflecting that this a common problem with pediatric ED studies. Values are unlikely to be missing at random, with sicker children more likely to have a complete set of observations documented. However, this would mean that the children expected to have missing values would be more likely to have a lower PEWS.

Neonates demonstrated a significantly higher MR-proADM than the remaining study population, but as they represent a small proportion of the study population, this was unlikely to change the overall conclusions of our study. Both the application of the phenotyping algorithm and measurement of biomarkers were performed retrospectively; it is therefore not possible to determine the impact of real-time results at the clinical decision-making stage. This would require a prospective randomized controlled trial, such as ongoing trial in adults in the United Kingdom comparing National Early Warning Score- 2 (NEWS2) versus an algorithm incorporating NEWS2 and procalcitonin for risk stratification in sepsis (32). Further interventional studies in different settings are required to determine if risk stratification using MR-proADM translates into improved outcomes.

## ACKNOWLEDGMENTS

We thank all of the children studied and their parents/carers for contributing to the study, and the medical and nursing staff at the Alder Hey Children’s National Health Service Foundation Trust Emergency Department for their contribution to the study. We thank the Salivary Procalcitonin for the Detection of Bacterial Infection in Children admitted to the Emergency Department study team for recruiting patients, collecting and curating data, and measuring procalcitonin in the laboratory (Dr. Adam Irwin, Mrs. Alison Grant, Mrs. Rhian Kenwright, Mrs. Christine Chesters, and Mr. Graham Jeffers).

## Supplementary Material


