# Supplementary figures and images for: Mid-Regional Pro-Adrenomedullin in Combination With Pediatric Early Warning Scores for Risk Stratification of Febrile Children Presenting to the Emergency Department: Secondary Analysis of a Nonprespecified United Kingdom Cohort Study*
Source: Pediatr Crit Care Med. 2022 Oct 14;23(12):980–9. doi: 10.1097/PCC.0000000000003075 (PMC9708078; doi:10.1097/PCC.0000000000003075)

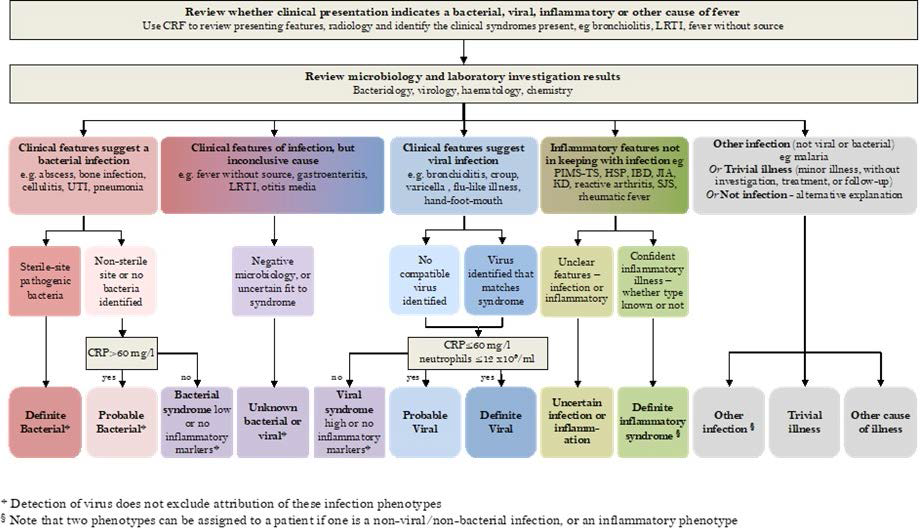

Supplement: Supplementary file 1 [file pcc-23-0980-s001.tif]

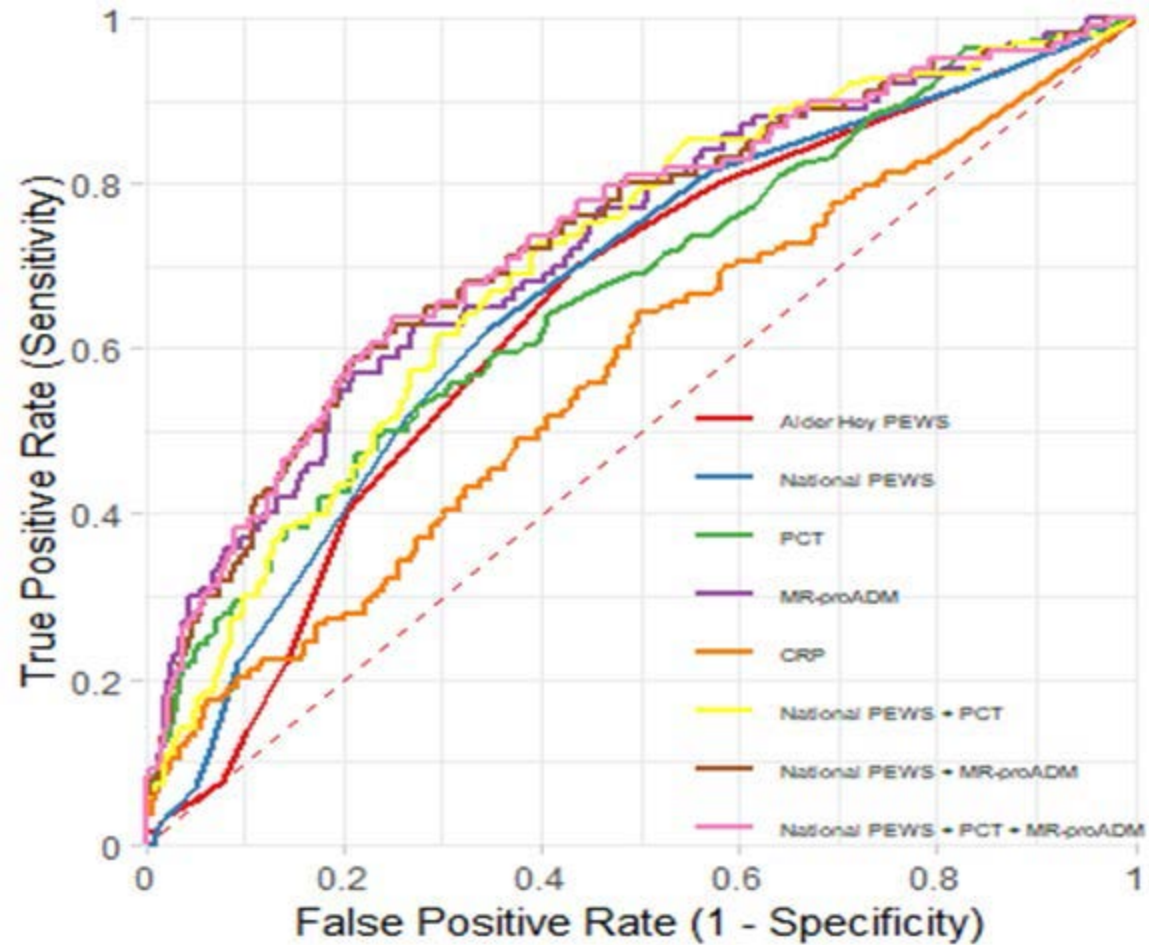

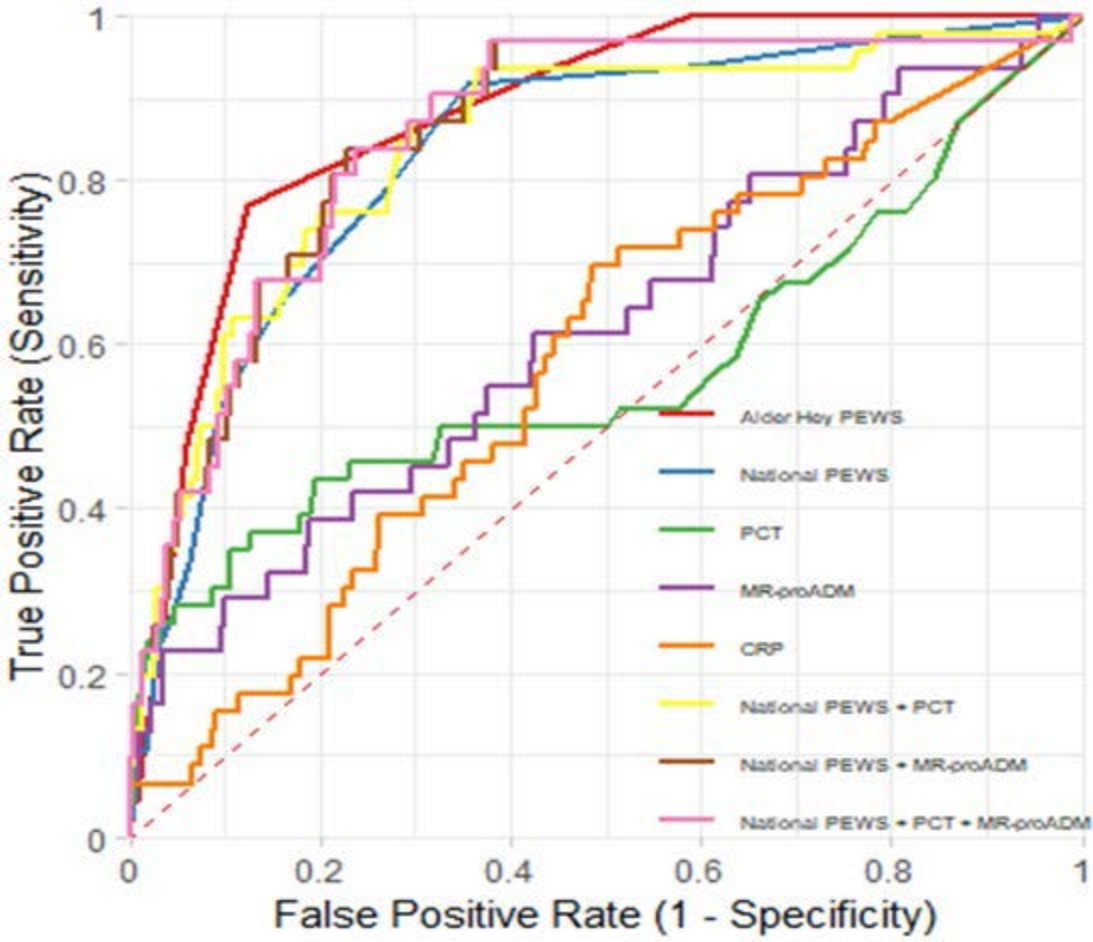

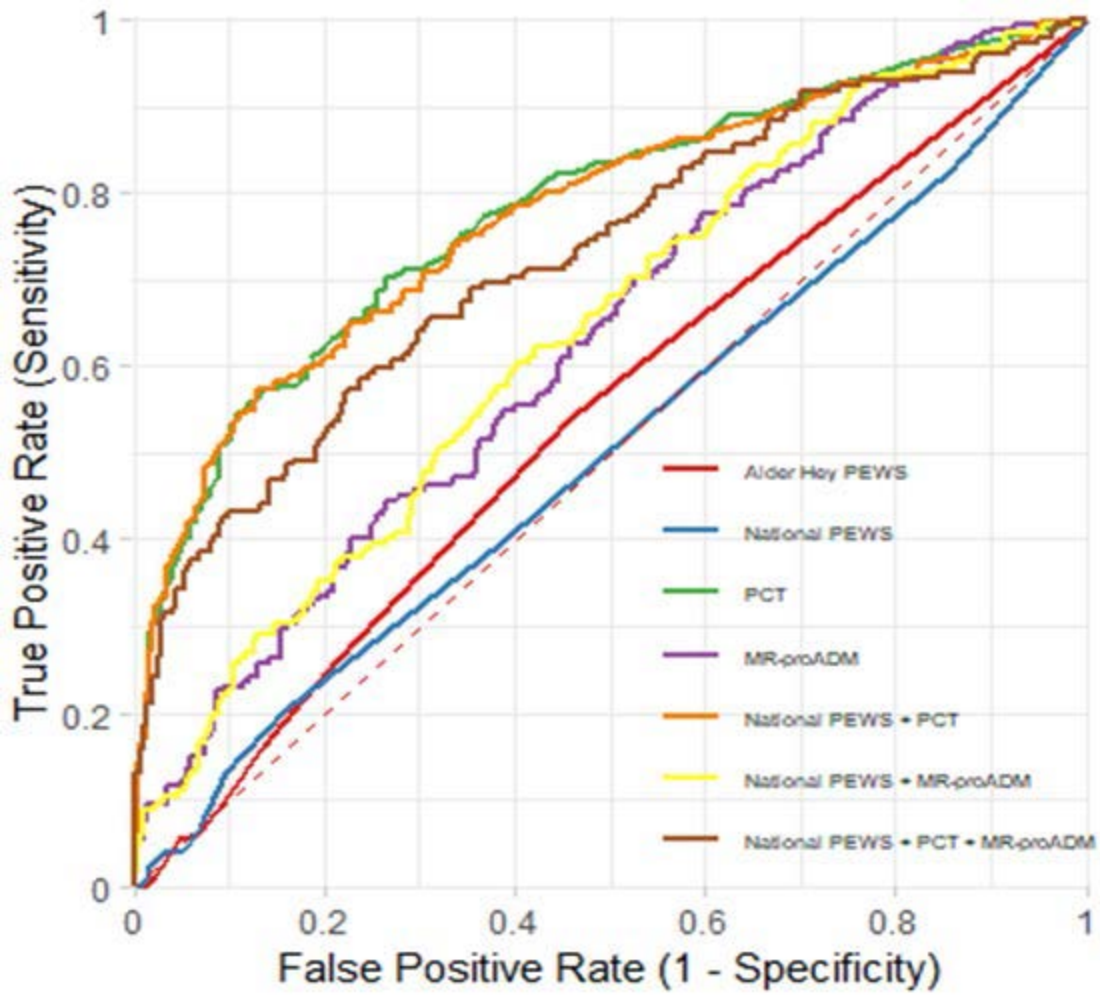

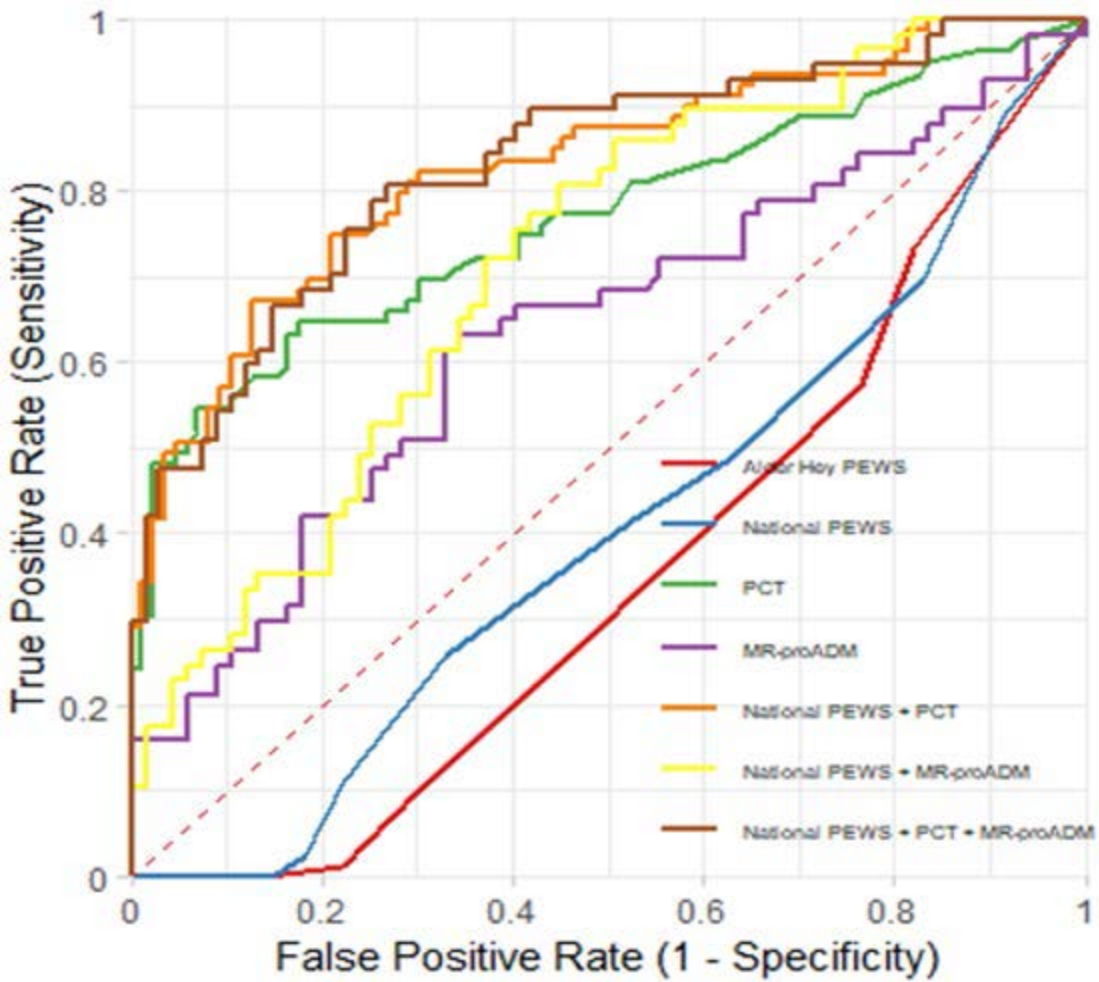

Supplement: Supplementary file 2 [file pcc-23-0980-s002.pdf]

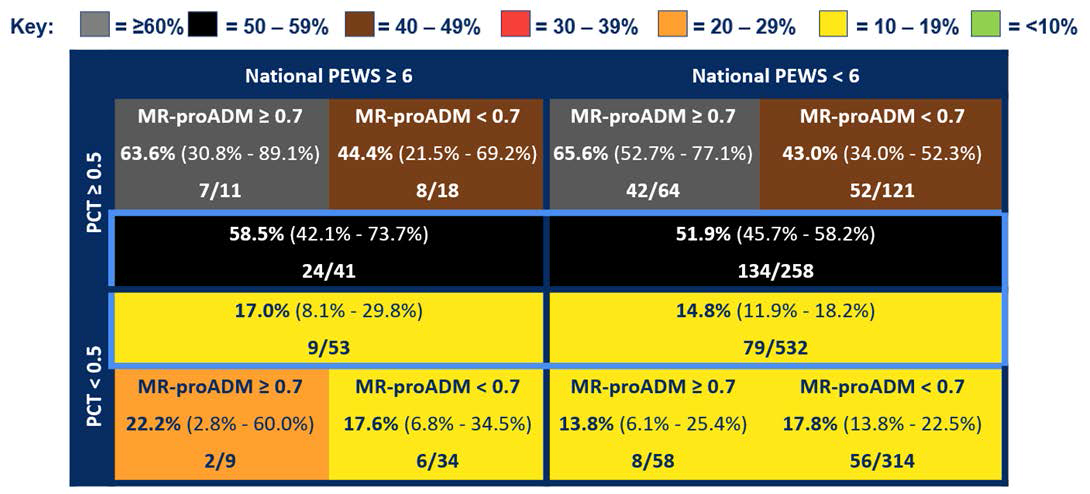

Supplement: Supplementary file 8 [file pcc-23-0980-s008.tif]
