# Supplementary material for: Mid-Regional Pro-Adrenomedullin in Combination With Pediatric Early Warning Scores for Risk Stratification of Febrile Children Presenting to the Emergency Department: Secondary Analysis of a Nonprespecified United Kingdom Cohort Study*
Source: Pediatr Crit Care Med. 2022 Oct 14;23(12):980–9. doi: 10.1097/PCC.0000000000003075 (PMC9708078; doi:10.1097/PCC.0000000000003075)
Supplement: Supplementary file 4 [file pcc-23-0980-s004.docx]

| Supplementary Table 1: Summary of patients with missing PEWS parameters | |
| --- | --- |
| PEWS Parameter | **Number of patients with missing values** |
| Blood Pressure | 1009 |
| Respiratory Rate | 218 |
| Capillary Refill Time | 192 |
| Oxygen Saturations | 159 |
| Heart Rate | 55 |
| Work of Breathing | 30 |
| Temperature | 12 |
| Mental State (AVPU) | 9 |
