# Supplementary material for: Mid-Regional Pro-Adrenomedullin in Combination With Pediatric Early Warning Scores for Risk Stratification of Febrile Children Presenting to the Emergency Department: Secondary Analysis of a Nonprespecified United Kingdom Cohort Study*
Source: Pediatr Crit Care Med. 2022 Oct 14;23(12):980–9. doi: 10.1097/PCC.0000000000003075 (PMC9708078; doi:10.1097/PCC.0000000000003075)
Supplement: Supplementary file 5 [file pcc-23-0980-s005.docx]

| **Supplementary Table 2: Diagnostic odds ratios, sensitivity, specificity, positive and negative predictive values, positive and negative likelihood ratios** | | | | | | | | |
| --- | --- | --- | --- | --- | --- | --- | --- | --- |
| **Scoring System and threshold** | **OR (95% CI)** | **Sensitivity; (95% CI)** | **Specificity; (95% CI)** | **Positive predictive value; (95% CI)** | **Negative predictive value; (95% CI)** | **Positive likelihood ratio (95% CI)** | **Negative likelihood ratio (95% CI)** | **Accuracy; (95% CI)** |
| **Critical Care Admission** | | | | | | | | |
| **AH PEWS ≥3** | 6.958 (8.099 - 35.510) | 0.812 (0.674 - 0.911) | 0.796 (0.772 - 0.830) | 0.144 (0.105 - 0.192) | 0.990 (0.981 - 0.995) | 3.992 (3.341 - 4.770) | 0.235 (0.131-0.425) | 0.797 (0.773 - 0.820) |
| **National PEWS ≥6** | 10.776 (5.907 - 19.656) | 0.521 (0.372 - 0.667) | 0.908 (0.890 - 0.925) | 0.194 (0.130 - 0.273) | 0.978 (0.967 - 0.986) | 5.684 (4.097 - 7.886) | 0.528 (0.393 - 0.709) | 0.893 (0.874 - 0.910) |
| **MR-proADM ≥0.7 nmol/L** | 2.638 (1.253 - 5.556) | 0.387 (0.218 - 0.578) | 0.807 (0.777 - 0.834) | 0.075 (0.040 - 0.128) | 0.970 (0.954 - 0.982) | 2.004 (1.257 - 3.194) | 0.760 (0.572 - 1.007) | 0.790 (0.760 - 0.818) |
| **PCT ≥0.5 ng/mL** | 2.075 (1.148 - 3.752) | 0.500 (0.349 - 0.651) | 0.675 (0.646 - 0.703) | 0.062 (0.040 - 0.092) | 0.969 (0.954 - 0.980) | 1.538 (1.137 - 2.079) | 0.741 (0.553 - 0.992) | 0.668 (0.639 - 0.695) |
| **CRP ≥20 mg/L** | 2.315 (1.222 - 4.386) | 0.696 (0.542 - 0.823) | 0.502 (0.472 - 0.533) | 0.055 (0.038 - 0.077) | 0.975 (0.959 - 0.987) | 1.400 (1.146 - 1.710) | 0.605 (0.389 - 0.940) | 0.511 (0.482 - 0.540) |
| **National PEWS ≥6 AND**  **MR-proADM ≥0.7 nmol/L** | 4.554 (1.294 - 16.025) | 0.077 (0.016 - 0.209) | 0.982 (0.972 - 0.989) | 0.130 (0.028 - 0.336) | 0.968 (0.956 - 0.978) | 4.281 (1.328 - 13.803) | 0.940 (0.858 - 1.029) | 0.951 (0.937 - 0.963) |
| **National PEWS ≥6 AND**  **PC T ≥ 0.5 ng/mL** | 9.123 (4.414 - 18.856) | 0.255 (0.139 - 0.403) | 0.964 (0.951 - 0.974) | 0.226 (0.123 - 0.362) | 0.969 (0.957-0.978) | 7.049 (3.972 - 12.506) | 0.773 (0.653 - 0.914) | 0.936 (0.920 - 0.949) |
| **National PEWS ≥AND MR-proADM ≥0.7 nmol/L AND PCT ≥0.5 ng/mL** | 8.795 (2.326 - 33.257) | 0.073 (0.015 - 0.199) | 0.991 (0.984 - 0.996) | 0.231 (0.050 - 0.538) | 0.967 (0.955 - 0.977) | 8.224 (2.352 - 28.764) | 0.935 (0.858 - 1.019) | 0.959 (0.946 - 0.969) |
| **Fluid Resuscitation** | | | | | | | | |
| **AH PEWS ≥3** | 2.655 (1.846 - 3.818) | 0.404 (0.324 - 0.488) | 0.797 (0.771 - 0.821) | 0.219 (0.171 - 0.273) | 0.905 (0.884 - 0.923) | 1.986 (1.577 - 2.502) | 0.748 (0.652 - 0.858) | 0.748 (0.722 - 0.773) |
| **National PEWS ≥6** | 2.720 (1.744 - 4.242) | 0.219 (0.155 - 0.295) | 0.906 (0.887 - 0.923) | 0.248 (0.176 - 0.332) | 0.892 (0.872 - 0.923) | 2.343 (1.635 - 3.359) | 0.861 (0.789 - 0.941) | 0.822 (0.799 - 0.843) |
| **MR-proADM ≥0.7 nmol/L** | 4.148 (2.666 - 6.455) | 0.450 (0.350 - 0.553) | 0.835 (0.805 - 0.862) | 0.283 (0.215 - 0.360) | 0.913 (0.888 - 0.934) | 2.732 (2.077 - 3.593) | 0.658 (0.550 - 0.789) | 0.787 (0.756 - 0.815) |
| **PCT ≥0.5 ng/mL** | 2.748 (1.910 - 3.956) | 0.544 (0.457 - 0.630) | 0.697 (0.667 - 0.726) | 0.201 (0.161 - 0.246) | 0.916 (0.894 - 0.935) | 1.797 (1.499 - 2.154) | 0.654 (0.542 - 0.789) | 0.678 (0.650 - 0.706) |
| **CRP ≥20 mg/L** | 1.616 (1.130 - 2.312) | 0.608 (0.523 - 0.689) | 0.510 (0.479 - 0.541) | 0.150 (0.122 - 0.181) | 0.902 (0.874 - 0.925) | 1.241 (1.073 - 1.436) | 0.768 (0.621 - 0.950) | 0.522 (0.493 - 0.551) |
| **National PEWS ≥6 AND**  **MR-proADM ≥0.7 nmol/L** | 4.284 (1.780 - 10.307) | 0.060 (0.026 - 0.115) | 0.985 (0.976 - 0.992) | 0.348 (0.164 - 0.572) | 0.889 (0.870 - 0.907) | 4.086 (1.776 - 9.454) | 0.954 (0.913 - 0.996) | 0.878 (0.858 - 0.897) |
| **National PEWS ≥6 AND**  **PC T ≥ 0.5 ng/mL** | 3.748 (20.45 - 6.869) | 0.119 (0.071 - 0.184) | 0.965 (0.952 - 0.976) | 0.321 (0.199 - 0.463) | 0.888 (0.868 - 0.906) | 3.421 (1.975 - 5.928) | 0.913 (0.859 - 0.971) | 0.863 (0.842 - 0.882) |
| **National PEWS ≥AND MR-proADM ≥0.7 nmol/L AND PCT ≥0.5 ng/mL** | 4.913 (1.584 - 15.243) | 0.037 (0.012 - 0.084) | 0.992 (0.985 - 0.997) | 0.385 (0.139 - 0.684) | 0.887 (0.867 - 0.905) | 4.769 (1.583 - 14.366) | 0.971 (0.939 - 1.004) | 0.882 (0.862 - 0.900) |
| **Definite and Probable Bacterial vs Definite and Probable Viral Infection** | | | | | | | | |
| **AH PEWS ≥3** | 1.309 (0.937 - 1.829) | 0.260 (0.207 - 0.318) | 0.789 (0.756 - 0.819) | 0.321 (0.258 - 0.388) | 0.735 (0.701 - 0.767) | 1.229 (0.865 - 1.019) | 0.939 (0.864 - 1.019) | 0.642 (0.610 - 0.673) |
| **National PEWS ≥6** | 1.552 (0.926 - 2.246) | 0.132 (0.093 - 0.179) | 0.905 (0.880 - 0.926) | 0.347 (0.254 - 0.450) | 0.731 (0.699 - 0.761) | 1.384 (0.936 - 2.045) | 0.960 (0.910 - 1.012) | 0.690 (0.660 - 0.720) |
| **MR-proADM ≥0.7 nmol/L** | 2.127 (1.438 - 3.145) | 0.324 (0.257 - 0.397) | 0.816 (0.777 - 0.851) | 0.415 (0.333 - 0.501) | 0.749 (0.709 - 0.787) | 1.761 (1.32 - 2.345) | 0.828 (0.742 - 0.924) | 0.675 (0.637 - 0.711) |
| **PCT ≥0.5 ng/mL** | 6.374 (4.623 - 8.788) | 0.642 (0.579 - 0.702) | 0.780 (0.746 - 0.813) | 0.530 (0.472 - 0.588) | 0.850 (0.818 - 0.878) | 2.922 (2.457 - 3.476) | 0.458 (0.386 - 0.545) | 0.742 (0.712 - 0.770) |
| **National PEWS ≥6 AND**  **MR-proADM ≥0.7 nmol/L** | 2.211 (0.905 - 5.403) | 0.036 (0.017 - 0.068) | 0.983 (0.970 - 0.992) | 0.450 (0.231 - 0.685) | 0.730 (0.699 - 0.759) | 2.168 (0.909 - 5.167) | 0.980 (0.955 - 1.006) | 0.724 (0.692 - 0.753) |
| **National PEWS ≥6 AND**  **PC T ≥ 0.5 ng/mL** | 3.951 (2.085 - 7.485) | 0.093 (0.061 - 0.136) | 0.975 (0.960 - 0.985) | 0.585 (0.421 - 0.737) | 0.737 (0.706 - 0.765) | 3.675 (2.008 - 6.726) | 0.930 (0.893 - 0.969) | 0.730 (0.700 - 0.758) |
| **National PEWS ≥AND MR-proADM ≥0.7 nmol/L AND PCT ≥0.5 ng/mL** | 4.787 (1.389 - 16.497) | 0.028 (0.011 - 0.057) | 0.994 (0.985 - 0.998) | 0.636 (0.308 - 0.891) | 0.732 (0.702 - 0.761) | 4.681 (1.382 - 15.852) | 0.978 (0.957 - 0.999) | 0.731 (0.701 - 0.760) |
| **Definite Bacterial vs Definite Viral Infection** | | | | | | | | |
| **AH PEWS ≥3** | 0.408 (0.215 - 0.775) | 0.256 (0.116 - 0.364) | 0.542 (0.427 - 0.646) | 0.328 (0.216 - 0.457) | 0.455 (0.361 - 0.552) | 0.560 (0.362 - 0.860) | 1.371 (1.095 - 1.717) | 0.409 (0.336 - 0.486) |
| **National PEWS ≥6** | 0.429 (0.184 - 0.998) | 0.110 (0.051 - 0.198) | 0.777 (0.679 - 0.856) | 0.300 (0.147 - 0.494) | 0.500 (0.416 - 0.584) | 0.491 (0.239 - 1.012) | 1.146 (1.004 - 1.309) | 0.466 (0.391 - 0.542) |
| **MR-proADM ≥0.7 nmol/L** | 2.273 (1.097 - 4.708) | 0.529 (0.390 - 0.660) | 0.672 (0.546 - 0.782) | 0.577 (0.432 - 0.713) | 0.625 (0.503 - 0.736) | 1.603 (1.051 - 2.444) | 0.705 (0.512 - 0.972) | 0.605 (0.513 - 0.691) |
| **PCT ≥0.5 ng/mL** | 4.975 (2.567 - 9.645) | 0.659 (0.543 - 0.761) | 0.721 (0.614 - 0.812) | 0.684 (0.567 - 0.786) | 0.697 (0.590 - 0.790) | 2.359 (1.621 - 3.432) | 0.474 (0.340 - 0.661) | 0.691 (0.614 - 0.760) |
| **National PEWS ≥6 AND**  **MR-proADM ≥0.7 nmol/L** | 0.664 (0.153 - 2.874) | 0.038 (0.008 - 0.108) | 0.943 (0.872 - 0.981) | 0.375 (0.085 - 0.755) | 0.525 (0.444 - 0.605) | 0.677 (0.167 - 2.741) | 1.019 (0.953 - 1.091) | 0.518 (0.439 - 0.596) |
| **National PEWS ≥6 AND**  **PC T ≥ 0.5 ng/mL** | 1.133 (0.380 - 3.380) | 0.085 (0.035 - 0.168) | 0.924 (0.849 - 0.969) | 0.500 (0.230 - 0.770) | 0.531 (0.451 - 0.610) | 1.122 (0.411 - 3.063) | 0.990 (0.906 - 1.081) | 0.529 (0.452 - 0.605) |
| **National PEWS ≥AND MR-proADM ≥0.7 nmol/L AND PCT ≥0.5 ng/mL** | 1.171 (0.161 - 8.513) | 0.026 (0.003 - 0.090) | 0.978 (0.923 - 0.997) | 0.500 (0.068 - 0.932) | 0.539 (0.460 - 0.617) | 1.167 (0.168 - 8.090) | 0.996 (0.950 - 1.045) | 0.538 (0.460 - 0.615) |
