# Supplementary material for: Mid-Regional Pro-Adrenomedullin in Combination With Pediatric Early Warning Scores for Risk Stratification of Febrile Children Presenting to the Emergency Department: Secondary Analysis of a Nonprespecified United Kingdom Cohort Study*
Source: Pediatr Crit Care Med. 2022 Oct 14;23(12):980–9. doi: 10.1097/PCC.0000000000003075 (PMC9708078; doi:10.1097/PCC.0000000000003075)
Supplement: Supplementary file 6 [file pcc-23-0980-s006.docx]

| Supplementary Table 3: Description of study population, Paediatric Early Warning Scores and biomarker data | |
| --- | --- |
| Demographics | |
| Number of children | 1183 |
| Male, n (%) | 654 (55.3) |
| Age in years, median (IQR) | 2.5 (4.8) |
| Pre-existing comorbidity, n (%) | 359 (30.3) |
| Multiple comorbidities, n (%) | 106 (9.0) |
| Outcomes | |
| Fluid resuscitation, n (%) | 146 (12.3) |
| Critical care admission, n (%) | 48 (4.1) |
| Definite bacterial infection, n (%) | 77 (6.5) |
| Probable bacterial infection, n (%) | 167 (14.1) |
| Definite viral infection, n (%) | 92 (7.8) |
| Probable viral infection, n (%) | 539 (45.6) |
| PEWS | |
| Alder Hey PEWS | |
| Missing, n (%) | 0 (0.0) |
| Range | 0 – 16 |
| Median (IQR) | 1 (2) |
| ≥3, n (%) | 270 (22.8) |
| National PEWS | |
| Missing, n (%) | 0 (0.0) |
| Range | 0 – 13 |
| Median, (IQR) | 2 (3) |
| ≥6, n (%) | 129 (10.9) |
| Biomarkers | |
| MR-proADM | |
| Total, n | 792 |
| Missing, n (%) | 391 (33.1) |
| Range (nmol/L) | 0 – 4.9 |
| Median (nmol/L), (IQR) | 0.51 (0.3) |
| ≥0.70 nmol/L, n (%) | 159 (20.1) |
| PCT | |
| Total, n | 1107 |
| Missing, n (%) | 76 (6.4) |
| Range (ng/mL) | 0 – 172 |
| Median (ng/mL), (IQR) | 0.23 (0.7) |
| ≥0.50 ng/mL, n (%) | 368 (33.2) |
| CRP | |
| Total, n | 1151 |
| Missing, n (%) | 32 (2.7) |
| Range (mg/L) | 4 – 483.5 |
| Median (mg/L), (IQR) | 20.4 (51.0) |
| ≥20 mg/L, n (%) | 581 (50.5) |
