# Supplementary material for: Mid-Regional Pro-Adrenomedullin in Combination With Pediatric Early Warning Scores for Risk Stratification of Febrile Children Presenting to the Emergency Department: Secondary Analysis of a Nonprespecified United Kingdom Cohort Study*
Source: Pediatr Crit Care Med. 2022 Oct 14;23(12):980–9. doi: 10.1097/PCC.0000000000003075 (PMC9708078; doi:10.1097/PCC.0000000000003075)
Supplement: Supplementary file 7 [file pcc-23-0980-s007.pdf]

Key: ■ = ≥50% ■ = 40 – 49% ■ = 30 – 39% ■ = 20 – 29% ■ = 10 – 19% ■ = <10%

4a.

| | National PEWS ≥ 6 | | National PEWS < 6 | |
|---|---|---|---|---|
| | MR-proADM ≥ 0.7 | MR-proADM < 0.7 | MR-proADM ≥ 0.7 | MR-proADM < 0.7 |
| PCT ≥ 0.5 | **38.5%** (13.9% - 68.4%) **5/13** | **16.0%** (4.5% - 36.1%) **4/25** | **31.2%** (21.1% - 42.7%) **24/77** | **12.6%** (7.7% - 19.0%) **19/151** |
| PCT ≥ 0.5 | **23.7%** (11.4% - 40.2%) **9/38** | | **18.9%** (14.0% - 24.6%) **43/228** | |
| PCT < 0.5 | **16.9%** (8.4% - 29.0%) **10/59** | | **8.0%** (5.7% - 10.9%) **37/461** | |
| PCT < 0.5 | MR-proADM ≥ 0.7 **30.0%** (6.7% - 65.2%) **3/10** | MR-proADM < 0.7 **14.3%** (5.9% - 27.2%) **7/49** | MR-proADM ≥ 0.7 **22.0%** (12.3% - 34.7%) **13/59** | MR-proADM < 0.7 **6.0%** (3.9% - 8.8%) **42/402** |

4b.

| | National PEWS ≥ 6 | | National PEWS < 6 | |
|---|---|---|---|---|
| | MR-proADM ≥ 0.7 | MR-proADM < 0.7 | MR-proADM ≥ 0.7 | MR-proADM < 0.7 |
| PCT ≥ 0.5 | **38.5%** (13.9% - 68.4%) **5/13** | **16.0%** (4.5% - 36.1%) **4/25** | **31.2%** (21.1% - 42.7%) **24/77** | **12.6%** (7.7% - 19.0%) **19/151** |
| PCT ≥ 0.5 | **23.7%** (11.4% - 40.2%) **9/38** | | **18.9%** (14.0% - 24.6%) **43/228** | |
| PCT < 0.5 | **16.9%** (8.4% - 29.0%) **10/59** | | **8.0%** (5.7% - 10.9%) **37/461** | |
| PCT < 0.5 | MR-proADM ≥ 0.7 **30.0%** (6.7% - 65.2%) **3/10** | MR-proADM < 0.7 **14.3%** (5.9% - 27.2%) **7/49** | MR-proADM ≥ 0.7 **22.0%** (12.3% - 34.7%) **13/59** | MR-proADM < 0.7 **6.0%** (3.9% - 8.8%) **42/402** |

4c.

| | National PEWS ≥ 6 | | National PEWS < 6 | |
|---|---|---|---|---|
| | MR-proADM ≥ 0.7 | MR-proADM < 0.7 | MR-proADM ≥ 0.7 | MR-proADM < 0.7 |
| PCT ≥ 0.5 | **53.8%** (25.1% - 80.8%) **7/13** | **32.0%** (14.9% - 53.5%) **8/25** | **54.5%** (42.8% - 65.9%) **42/77** | **34.4%** (26.9% - 42.6%) **52/151** |
| PCT ≥ 0.5 | **39.5%** (24.0% - 56.6%) **15/38** | | **41.2%** (34.8% - 47.9%) **94/228** | |
| PCT < 0.5 | **13.6%** (6.0% - 25.0%) **8/59** | | **13.9%** (10.9% - 17.4%) **64/461** | |
| PCT < 0.5 | MR-proADM ≥ 0.7 **20.0%** (2.5% - 55.6%) **2/10** | MR-proADM < 0.7 **12.2%** (4.6% - 24.8%) **6/49** | MR-proADM ≥ 0.7 **13.6%** (6.0% - 25.0%) **8/59** | MR-proADM < 0.7 **13.9%** (10.7% - 17.7%) **56/402** |
