# Supplementary material for: Mid-Regional Pro-Adrenomedullin in Combination With Pediatric Early Warning Scores for Risk Stratification of Febrile Children Presenting to the Emergency Department: Secondary Analysis of a Nonprespecified United Kingdom Cohort Study*
Source: Pediatr Crit Care Med. 2022 Oct 14;23(12):980–9. doi: 10.1097/PCC.0000000000003075 (PMC9708078; doi:10.1097/PCC.0000000000003075)
Supplement: Supplementary file 9 [file pcc-23-0980-s009.docx]

# **Supplementary Figure Legends**

Supplementary Figure 1: Phenotyping flowchart based on PERFORM algorithm, reproduced with permission from Professor Enitan D. Carrol. (Nijman RG et al.: Management of Children With Fever at Risk for Pediatric Sepsis: A Prospective Study in Pediatric Emergency Care. Front Pediatr 2020; 8:548154)

Supplementary Figure 2: Receiver operating curves for biomarkers, clinical scores and combined risk stratification

Supplementary Figure 2a: Receiver operating curve for fluid resuscitation

Supplementary Figure 2b: Receiver operating curve for critical care admission

Supplementary Figure 2c: Receiver operating curve for definite and probable bacterial infection vs definite and probable viral infection

Supplementary Figure 2d: Receiver operating curve for definite bacterial infection vs definite viral infection

Supplementary Figure 3: Patient recruitment flowchart

Supplementary Figure 4: Risk stratification by Mid-Regional Pro-Adrenomedullin (MR-proADM), Procalcitonin (PCT) and National Paediatric Early Warning Score (PEWS) excluding patients with missing data points

Values stated are absolute percentages (with 95% confidence intervals) with frequencies of patients with each given outcome over total number of patients meeting stated MR-proADM, PCT and National PEWS criteria. All groups for risk stratification for this supplementary figure include only patients with an available PCT, PEWS and MR-proADM with no missing values.

Supplementary Figure 4a: Risk stratification for critical care admission

Supplementary Figure 4b: Risk stratification for fluid resuscitation

Supplementary Figure 4c: Risks stratification for definite and probable bacterial vs definite and probable viral infection

Supplementary Figure 5: Risk stratification figure excluding patients not meeting criteria for definite/probable bacterial or viral infection

Comparison of definite and probable bacterial compared with definite and probable viral infection, excluding all patients who fit into other PERFORM phenotyping categories. Values stated are absolute percentages (with 95% confidence intervals) with frequencies of patients with each given outcome over total number of patients meeting stated MR-proADM, PCT and National PEWS criteria. Groups for risk stratification highlighted in light blue include all patients with a PCT and PEWS value available, with the outer sub-stratification boxes only including patients with an available PCT, PEWS and MR-proADM with no missing values
