# Supplementary material for: Mid-Regional Pro-Adrenomedullin in Combination With Pediatric Early Warning Scores for Risk Stratification of Febrile Children Presenting to the Emergency Department: Secondary Analysis of a Nonprespecified United Kingdom Cohort Study*
Source: Pediatr Crit Care Med. 2022 Oct 14;23(12):980–9. doi: 10.1097/PCC.0000000000003075 (PMC9708078; doi:10.1097/PCC.0000000000003075)
Supplement: Supplementary file 7 [file pcc-23-0980-s003.docx]

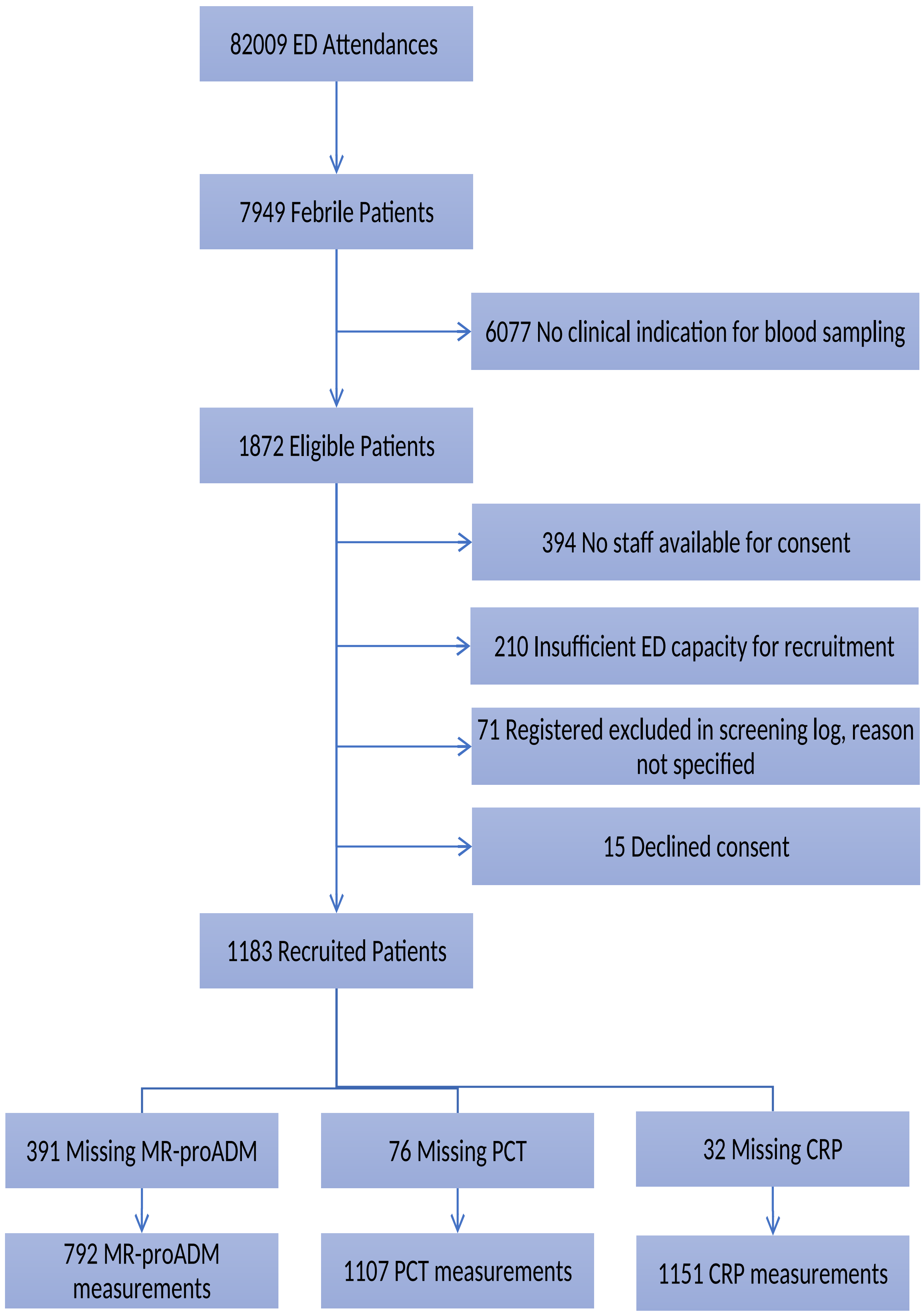
82009 ED Attendances
7949 Febrile Patients
6077 No clinical indication for blood sampling
1872 Eligible Patients
394 No staff available for consent
210 Insufficient ED capacity for recruitment
71 Registered excluded in screening log, reason not specified
15 Declined consent
1183 Recruited Patients
391 Missing MR-proADM
76 Missing PCT
32 Missing CRP
792 MR-proADM measurements
1107 PCT measurements
1151 CRP measurements
